# Incidence and risk factors for venous and arterial thromboses in hospitalized patients with coronavirus disease 2019: data on 4014 patients from a tertiary center registry

**DOI:** 10.3325/cmj.2022.63.16

**Published:** 2022-02

**Authors:** Ivana Jurin, Marko Lucijanić, Nevenka Piskač Živković, Kristina Lalić, Anamarija Zrilić Vrkljan, Linda Malnar Janeš, Ivona Kovačević, Tomislav Čikara, Anica Sabljić, Nikolina Bušić, Gorana Vukorepa, Irzal Hadžibegović, Ivica Lukšić, Bruno Baršić

**Affiliations:** 1 Primary Respiratory and Intensive Care Center, University Hospital Dubrava, Zagreb, Croatia; 2 Cardiology Department, University Hospital Dubrava, Zagreb, Croatia; 3 Hematology Department, University Hospital Dubrava, Zagreb, Croatia; 4 University of Zagreb, School of Medicine, Zagreb, Croatia; 5 Pulmology Department, University Hospital Dubrava, Zagreb, Croatia; 6 Endocrinology Department, University Hospital Dubrava, Zagreb, Croatia; 7 Neurology Department, University Hospital Dubrava, Zagreb, Croatia; 8 Faculty of Dental Medicine and Healthcare, Josip Juraj Strossmayer University, Osijek, Croatia; 9 Department of Maxillofacial Surgery, University Hospital Dubrava, Zagreb, Croatia

## Abstract

**Aim:**

To evaluate the burden and predictors of thromboembolic complications in a large real-life cohort of hospitalized patients with established coronavirus disease 2019 (COVID-19).

**Methods:**

We retrospectively reviewed the records of 4014 consecutive adult patients admitted to a tertiary-level institution because of COVID-19 from March 2020 to March 2021 for the presence of venous and arterial thrombotic events.

**Results:**

Venous-thromboembolic (VTE) events were present in 5.3% and arterial thrombotic events in 5.8% patients. The majority of arterial thromboses occurred before or on the day of admission, while the majority of VTE events occurred during hospitalization. The majority of both types of events occurred before intensive care unit (ICU) admission, although both types of events were associated with a higher need for ICU use and prolonged immobilization. In multivariate logistic regression, VTE events were independently associated with metastatic malignancy, known thrombophilia, lower mean corpuscular hemoglobin concentration, higher D-dimer, lower lactate dehydrogenase, longer duration of disease on admission, bilateral pneumonia, longer duration of hospitalization, and immobilization for at least one day. Arterial thromboses were independently associated with less severe COVID-19, higher Charlson comorbidity index, coronary artery disease, peripheral artery disease, history of cerebrovascular insult, aspirin use, lower C reactive protein, better functional status on admission, ICU use, immobilization for at least one day, absence of hyperlipoproteinemia, and absence of metastatic malignancy.

**Conclusion:**

Among hospitalized COVID-19 patients, venous and arterial thromboses differ in timing of presentation, association with COVID-19 severity, and other clinical characteristics.

An increasing pool of evidence accumulating since the early days of the coronavirus disease 2019 (COVID-19) pandemic shows that severe acute respiratory syndrome coronavirus 2 (SARS-CoV-2) infection induces pro-thrombotic state. Although the disease presents dominantly with respiratory symptoms, resulting in acute respiratory distress syndrome in a subset of patients (1-4), high frequencies of venous and arterial thromboses were observed (5). SARS-CoV-2 endothelial tropism and damage to the vasculature of the lungs, heart, extremities, and brain have been recognized as the key part of disease pathophysiology (6-9). In addition, coagulopathy due to upregulation of inflammatory prothrombotic proteins, platelet activation, and immobilization of patients due to functional deterioration or connection to the oxygen source further favor the development of thrombotic incidents. Vascular complications occur mostly in severe cases and are often associated with multiorgan failure and higher mortality. In some patients, they can also be the dominant clinical presentation. The most frequently noted thrombotic events are pulmonary embolism (PE) and deep vein thrombosis (DVT) (10), followed by stroke, acute limb ischemia, and acute coronary syndromes (11,12), developing despite the use of pharmacologic thromboprophylaxis in hospitalized COVID-19 patients.

Vascular endothelial injury is not COVID-19 specific, as similar response was described in other acute infectious diseases (13,14). However, the large scale of the COVID-19 pandemic and associated strain on the health care system result in a substantial number of patients urgently presenting to the hospital or being under an increased risk of development of thromboembolic complications. Due to importance of this issue and scarcity of regional data that may help guide the medical care of COVID-19 patients, studies on this issue are highly needed. Thus, the aim of this study was to evaluate the burden of thromboembolic complications and their predictors in a large real-life cohort of hospitalized patients with established COVID-19.

## Methods

The presented data are part of the hospital's Registry project, which gathers clinical and laboratory data, as well as data on the outcomes of all hospitalized COVID-19 patients treated in our institution during the COVID-19 pandemic. Data were obtained from electronic and written medical records. In March 2020, University Hospital Dubrava became a dedicated COVID-19 tertiary hospital center, serving 1.8 million inhabitants (the City of Zagreb and 6 surrounding counties). From March 2020 to March 2021, there were 4102 hospitalizations of 4014 individual patients. We retrospectively reviewed the records of 4014 consecutive adults at the time of their index hospitalization for acute COVID-19. All patients had a positive polymerase chain reaction or antigen COVID-19 test before hospital admission. Patients were treated according to the contemporary guidelines. The majority (85.9%) received pharmacologic thromboprophylaxis with low molecular weight heparins (LMWH). The study was approved by the Institutional Review board.

COVID-19 disease severity on admission was graded according to the World Health Organization (WHO) and national guidelines as mild, moderate, severe, and critical (15,16). The severity of COVID-19 symptoms was quantified by using the modified early warning score (MEWS). The functional status at admission was classified by using the Eastern Cooperative Oncology Group (ECOG) system. Comorbidities were assessed as individual entities and were summarized by using the Charlson Comorbidity Index. Venous and arterial thrombotic events were documented during the hospitalization and based on objective imaging and laboratory methods. Deep venous thrombosis (DVT), pulmonary embolism (PE), and other site venous thromboses were considered as venous thromboembolic events (VTE). Myocardial infarction, cerebrovascular insult, peripheral artery embolization, and mesenterial artery thromboses were considered as arterial thrombotic events.

### Statistical methods

The normality of distribution was tested using the Kolmogorov-Smirnov test. Numerical variables were presented as median and interquartile range (IQR) and categorical variables as frequencies and percentages. Differences in numerical variables between subgroups were assessed by using the Mann-Whitney U test. Differences in categorical variables between subgroups were assessed with the Χ^2^ test. Receiver operating characteristic (ROC) curve analysis was used to define optimal D-dimer cut-off levels for thrombosis. Associations of clinical parameters with the occurrence of venous and arterial thrombosis were assessed by using the logistic regression analysis. For the assessment of independent associations of specific comorbidities with survival, a model was built by using the backward approach, with *P* < 0.05 and *P* > 0.1 criteria for variable inclusion and removal, respectively. *P* values <0.05 were considered statistically significant. All analyses were performed with MedCalc statistical software, version 20.006 (MedCalc Software Ltd, Ostend, Belgium).

## Results

### Patients’ characteristics and venous and arterial thrombotic events

We reviewed the records of 4014 hospitalized COVID-19 patients (2256 or 56.2% men). The median age was 74 years IQR (64-82), and median Charlson Comorbidity Index was 4 IQR (3-6). A total of 3359 (83.7%) patients presented with severe or critical COVID-19 on admission. Overall, 913 (22.7%) required intensive care unit treatment, 771 (19.2%) required high-flow oxygen therapy, and 675 (16.8%) required mechanical ventilation.

**Figure 1:**
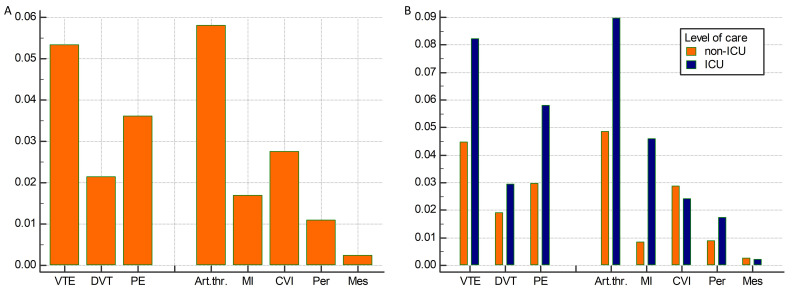
Frequencies of venous and arterial thrombotic events in hospitalized coronavirus disease 2019 (COVID-19) patients in (**A**) an overall cohort and (**B**) stratified to the level of intensive care required. VTE – venous thromboembolism; PE – pulmonary embolism; DVT – deep vein thrombosis; Art. Thr. – arterial thrombosis; MI – myocardial infarction; CVI – cerebrovascular insult; Per – peripheral artery embolization; Mes – mesenterial artery thromboses; ICU – intensive care unit.

Venous thromboembolic events were present in 214 (5.3%) patients, with DVT in 86 (2.1%) and PE in 145 (3.6%). Arterial thrombotic events were present in 233 (5.8%) patients, with myocardial infarction in 68 (1.7%), cerebrovascular insult in 111 (2.8%), peripheral embolization in 44 (1.1%), and mesenterial thrombosis in 10 (0.2%) (Figure 1A). The majority of arterial thrombotic events occurred before or on the day of admission (64.8% before or on the day of admission vs 35.2% during hospital stay). In contrast, the majority of venous thromboembolic events occurred during hospital stay (38.4% before or on the day of admission vs 61.6% during hospital stay). The median time of post-admission event occurrence/detection was 5.5 days (interquartile range [IQR] 3-10) for arterial and 11 days (IQR 4-21) for venous thrombotic events. Out of 214 venous events, 21 (9.8%) occurred after intensive care unit admission. Out of 233 arterial events, 23 (9.9%) occurred after intensive care unit admission.

### Factors associated with venous thromboses

Considering general characteristics, comorbidities, and drugs in chronic therapy, venous thromboses were significantly associated with a fewer drugs in chronic therapy (median 4 vs 5), were less frequent among patients with metabolic syndrome (VTE 3.6% vs 5.8%) and congestive heart failure (VTE 3.5% vs 5.7%), but were more frequent among patients with a history of VTE (VTE 14.5% vs 4.9%), known thrombophilia (23.8% vs 5.2%), and metastatic malignant disease (VTE 8.6% vs 5.1%) (Table 1). Considering laboratory parameters on admission, venous thromboses were significantly associated with higher white blood cells (WBC; median 9.2 vs 7.9 × 10^9^/L), lower mean corpuscular hemoglobin concentration (MCHC; median 330 vs 333 g/L), higher platelets (median 245 vs 219 × 10^9^/L), higher D-dimer (median 4.18 vs 1.36 mg/L fibrinogen equivalent unit [FEU]), higher lactate dehydrogenase (LDH; median 330 vs 335 U/L but average 448 vs 389 U/L), lower albumin (median 30 vs 32 g/L), and lower prothrombin time (PT; median 95 vs 100%) (Table 2).

**Table 1:** The relationship of venous and arterial thromboses with demographic characteristics, comorbidities, and selected drugs

	Overall (N = 4014)†	OR with 95% CI for venous thrombosis; P	OR with 95% CI for arterial thrombosis; P
**Age** (years)	74 (64-82)	0.99 (0.98-1.0); 0.317	1.02 (1.01-1.031); <0.001
**Male sex**	2256 (56.2)	0.78 (0.59-1.03); 0.083	0.86 (0.66-1.13); 0.279
**Charlson Comorbidity Index**	4 (3-6)	0.96 (0.91-1.01); 0.152	1.19 (1.13-1.24); <0.001
**Alcohol use**	218 (5.4)	1.23 (0.7-2.16); 0.462	1.12 (0.64-1.96); 0.689
**Smoking**	231 (5.8)	1.25 (0.73-2.15); 0.419	1.88 (1.19-2.96); 0.006
**Number of drugs in chronic therapy**	5 (2-8)	0.94 (0.91-.98); 0.002	1.06 (1.03-1.09); <0.001
**Arterial hypertension**	2771 (69)	0.75 (0.57-1.0); 0.054	1.83 (1.32-2.54); <0.001
**Diabetes mellitus**	1201 (29.9)	0.74 (0.54-1.02); 0.066	1.51 (1.15-1.98); 0.003
**Hyperlipoproteinemia**	954 (23.8)	0.7 (0.49-1.0); 0.051	1.53 (1.15-2.04); 0.003
**Obesity**	1069 (26.6)	0.95 (0.69-1.3); 0.752	0.74 (0.54-1.02); 0.066
**Metabolic syndrome**	799 (19.9)	0.62 (0.41-0.92); 0.018	1.33 (0.97-1.81); 0.073
**Congestive heart failure**	649 (16.2)	0.61 (0.39-0.95); 0.028	1.93 (1.42-2.61); <0.001
**Atrial fibrillation**	721 (18)	0.73 (0.49-1.09); 0.124	1.84 (1.36-2.48); <0.001
**Coronary artery disease**	613 (15.3)	0.65 (0.42-1.02); 0.060	2.98 (2.24-3.99); <0.001
**Peripheral artery disease**	281 (7)	0.85 (0.48-1.51); 0.586	4.72 (3.39-6.59); <0.001
**History of myocardial infarction**	366 (9.1)	0.58 (0.32-1.05); 0.070	2.5 (1.77-3.54); <0.001
**History of cerebrovascular insult**	469 (11.7)	0.99 (0.65-1.54); 0.999	3.81 (2.83-5.13); <0.001
**History of VTE**	193 (4.8)	3.31 (2.16-5.08); <0.001	0.99 (0.52-1.83); 0.949
**Chronic kidney disease**	498 (12.4)	0.67 (0.42-1.09); 0.109	1.5 (1.06-2.14); 0.024
**Chronic hemodialysis**	76 (1.9)	0.47 (0.12-1.95); 0.301	0.43 (0.11-1.78); 0.246
**GERD/Ulcer disease**	566 (14.1)	1.29 (0.89-1.86); 0.169	1.41 (0.99-1.98); 0.050
**Inflammatory bowel disease**	46 (1.1)	1.24 (0.38-4.04); 0.718	2.47 (1.04-5.89); 0.041
**Chronic liver disease**	110 (2.7)	1.03 (0.44-2.36); 0.954	0.76 (0.31-1.9); 0.568
**Liver cirrhosis**	49 (1.2)	1.16 (0.36-3.76); 0.804	0.33 (0.05-2.44); 0.280
**Epilepsy**	112 (2.8)	1.19 (0.55-2.59); 0.661	0.59 (0.22-1.63); 0.311
**Mental retardation**	45 (1.1)	1.27 (0.39-4.14); 0.689	0.36 (0.05-2.67); 0.322
**Schizophrenia**	60 (1.5)	2 (0.85-4.7); 0.112	0.56 (0.13-2.29); 0.416
**Dementia**	829 (20.7)	0.94 (0.66-1.32); 0.703	0.99 (0.72-1.38); 0.984
**Active malignant disease**	429 (10.7)	1.33 (0.88-1.99); 0.165	0.55 (0.33-0.95); 0.033
**Metastatic malignant disease**	280 (7)	1.75 (1.12-2.73); 0.013	0.39 (0.19-0.85); 0.018
**History of malignant disease**	718 (17.9)	1.27 (0.91-1.78); 0.158	0.59 (0.39-0.88); 0.011
**Thyroid disease**	371 (9.2)	1.13 (0.72-1.79); 0.590	0.92 (0.57-1.47); 0.721
**Autoimmune/rheumatic disease**	174 (4.3)	0.85 (0.41-1.75); 0.660	1.21 (0.66-2.21); 0.529
**Asthma**	119 (3)	0.61 (0.22-1.67); 0.336	0.71 (.029-1.74); 0.450
**COPD**	286 (7.1)	0.63 (0.33-1.19); 0.155	1.17 (0.72-1.89); 0.529
**Transplanted organ**	43 (1.1)	-	0.38 (0.05-2.8); 0.345
**Trauma/surgery 1 month prior or during hospitalization**	526 (13.1)	0.87 (0.56-1.33); 0.537	1.69 (1.21-2.37); 0.002
**Known thrombophilia**	21 (0.5)	5.66 (2.05-15.59); <0.001	2.73 (0.79-9.32); 0.109
**Anticoagulant therapy**	585 (14.6)	0.88 (0.58-1.32); 0.526	1.11 (0.77-1.6); 0.561
**Aspirin**	765 (19.1)	0.97 (0.69-1.39); 0.884	2.67 (2.02-3.53); <0.001
**Steroids prior to admission**	489 (12.2)	1.09 (0.72-1.64); 0.679	0.82 (0.53-1.26); 0.366
**Antipsychotics**	413 (10.3)	0.84 (0.52-1.36); 0.486	0.71 (0.44-1.17); 0.186
**Antidepressants**	288 (7.2)	0.83 (0.47-1.47); 0.522	1.16 (0.71-1.88); 0.551
**Active chemotherapy**	101 (2.5)	1.55 (0.74-3.23); 0.244	0.66 (0.24-1.81); 0.425
**Statin**	962 (24)	0.76 (0.54-1.08); 0.127	1.86 (1.41-2.46); <0.001
**Hormonal therapy**	92 (2.3)	1.02 (0.41-2.54); 0.964	0.54 (0.17-1.72); 0.296

*Abbreviations: OR – odds ratio; CI – confidence interval; VTE – venous thromboembolism; GERD – gastroesophageal reflux disease; COPD – chronic obstructive pulmonary disease.

†n (%) or median (interquartile range).

**Table 2:** The relationship of venous and arterial thromboses with laboratory parameters on admission*

	Overall (N = 4014); median (interquartile range)	OR with 95% CI for venous thrombosis; P	OR with 95% CI for arterial thrombosis; P
**Interleukin-6** (pg/mL)	53.4 (20.9-121.8)	0.99 (0.99-1.0); 0.303	0.99 (0.99-1.0); 0.279
**Procalcitonin** (ng/mL)	21.5 (0.09-0.76)	0.94 (0.88-1.0); 0.063	1.0 (0.98-1.02); 0.608
**White blood count** ( × 10^9^/L)	8 (5.7-11.2)	1.02 (1.01-1.04); 0.002	1.01 (1.0-1.03); 0.012
**Hemoglobin** (g/L)	128 (113-141)	0.99 (0.99-1.0); 0.204	1.0 (0.99-1.0); 0.329
**Mean corpuscular volume** (fL)	88.9 (85.6-92.2)	1.01 (0.99-1.03); 0.449	0.99 (0.97-1.01); 0.489
**Mean corpuscular hemoglobin concentration** (g/L)	333 (324-340)	0.98 (0.97-0.99); <0.001	0.98 (0.97-1.0); 0.052
**RDW** (%)	14.1 (13.4-15.2)	1.05 (0.98-1.11); 0.153	1.03 (0.96-1.09); 0.391
**Platelets** ( × 10^9^/L)	220 (163-296)	1.0 (1.0-1.0); 0.005	0.99 (0.99-1.0); 0.762
**C-reactive protein** (mg/L)	88.2 (39.5-150.8)	1.0 (0.99-1.0); 0.970	0.99 (0.99-0.99); <0.001
**Ferritin** (μg/L)	711 (386-1290)	1.0 (0.99-1.0); 0.691	0.99 (0.99-1.0); 0.717
**D-dimer** (mg/L FEU)	1.42 (0.73-3.58)	1.61 (1.45-1.79); <0.001	1.12 (1.01-1.25); 0.035
**Estimated glomerular filtration rate** (mL/min/1.73m^2^)	71.6 (45.8-90.4)	1.01 (0.99-1.01); 0.054	0.99 (0.99-1.0); 0.105
**Lactate dehydrogenase** (U/L)	335 (248-453)	1.0 (1.0-1.0); 0.040	1.0 (0.99-1.0); 0.235
Aspartate transaminase (U/L)	41 (28-64)	1.0 (1.0-1.0); 0.063	1.0 (0.99-1.0); 0.375
**Alanine transaminase** (U/L)	31 (19-52)	1.0 (0.99-1.0); 0.088	1.0 (0.99-1.0); 0.526
Gamma-glutamyltransferase (U/L)	42 (24-81)	0.99 (0.99-1.0); 0.052	0.99 (0.99-1.0); 0.068
**Alkaline phosphatase** (U/L)	72 (56-97)	0.99 (0.99-1.0); 0.307	0.99 (0.99-1.0); 0.404
**Total bilirubin** (μmol/L)	11.4 (8.6-15.9)	1.0 (0.99-1.0); 0.299	0.98 (0.97-1.0); 0.170
**Albumin** (g/L)	32 (28-35)	0.95 (0.92-0.98); <0.001	1.02 (0.98-1.06); 0.328
**Prothrombin time** (%)	100 (89-109)	0.98 (0.97-0.99); <0.001	0.99 (0.98-1.0); 0.406

*Abbreviations: OR – odds ratio; CI – confidence interval; FEU – fibrinogen equivalent unit.

Considering COVID-19 severity- and hospitalization-related parameters, venous thromboses were significantly more frequent among patients with longer duration of COVID-19 on admission (median 7 vs 5 days), higher COVID-19 severity (VTE 5.9% vs 2.6% among severe or critical vs mild or moderate severity), pneumonia (VTE 5.7% vs 2.5%, and bilateral pneumonia (VTE 6.1% vs 4%), and respiratory insufficiency requiring oxygen supplementation therapy (VTE 5.9% vs 2.8%). Venous thromboses were significantly associated with longer duration of hospital stay (median 15 vs 10 days), intensive care unit level of care (VTE 8.2% vs 4.5%), immobilization without bathroom privileges for at least 1 day (VTE 6.8% vs 1.8%), prolonged immobilization ≥7 days (VTE 7% vs 4.1%), and intensified corticosteroid therapy (VTE 8.4% vs 4.1%) (Table 3). They were not significantly associated with age, sex, mechanical ventilation, bleeding events, or arterial thromboses.

**Table 3:** The relationship of venous and arterial thromboses with coronavirus disease-2019 (COVID-19) severity and hospitalization-related parameters*

	Overall (N = 4014)^†^	OR with 95% CI for venous thrombosis	OR with 95 CI% for arterial thrombosis
**Origin of referral**			
**home**	1477 (36.8)	Reference category	Reference category
**nursing home**	493 (12.3)	0.76 (0.45-1.29); 0.313	OR 1.25 (0.76-2.09); 0.381
**Other hospital**	2044 (50.9)	1.32 (0.98-1.78); 0.069	2.25 (1.64-3.09); <0.001
**Day of disease on admission**	5 (1-9)	1.05 (1.03-1.07); <0.001	0.96 (0.93-0.98); 0.002
**ECOG status on admission**	3 (1-4)	1.01 (0.91-1.12); 0.895	1.24 (1.12-1.39); <0.001
**Pneumonia**	3531 (88)	2.38 (1.32-4.29); 0.004	0.73 (0.51-1.06); 0.099
**Bilateral pneumonia**	2600 (64.8)	1.57 (1.15-2.14); 0.005	0.78 (0.59-1.02); 0.069
**Oxygen therapy**	3265 (81.3)	2.17 (1.38-3.44); <0.001	0.76 (0.56-1.05); 0.099
**MEWS score**	2 (1-4)	1.08 (1.02-1.17); 0.013	0.87 (0.81-0.94); <0.001
**COVID-19 severity**			
**mild**	449 (11.2)	Reference category	Reference category
**moderate**	206 (5.1)	1.54 (0.58-4.12); 0.385	0.99 (0.54-1.84); 0.990
**severe**	2761 (68.8)	2.54 (1.33-4.86); 0.005	0.69 (0.47-1.01); 0.061
**critical**	598 (14.9)	3.66 (1.83-7.33); <0.001	0.6 (0.36-1.0); 0.051
**Other infection on admission**	587 (14.6)	1.32 (0.92-1.89); 0.127	1.61 (1.16-2.23); 0.005
**Length of hospitalization (days)**	10 (6-16)	1.05 (1.04-1.06); <0.001	1.01 (0.99-1.2); 0.063
**Intensive care unit**	913 (22.7)	1.91 (1.43-2.55); <0.001	1.92 (1.46-2.54); <0.001
**High-flow oxygen therapy**	771 (19.2)	1.38 (0.99-1.89); 0.053	0.98 (0.69-1.37); 0.897
**Mechanical ventilation**	675 (16.8)	1.15 (0.8-1.63); 0.451	1.57 (1.15-2.16); 0.005
**Immobilization >1 day**	2833 (70.6)	4.04 (2.56-6.37); <0.001	9.04 (4.92-16.6); <0.001
**Immobilization ≥7 days**	1769 (44.1)	1.77 (1.34-2.34); <0.001	1.74 (1.33-2.28); <0.001
**Current lower limb paralysis**		1.12 (0.83-1.49); 0.452	3.09 (2.37-4.05); <0.001
**Venous thromboembolism**	215 (5.3)	-	0.96 (0.53-1.75); 0.899
**Pulmonary embolism**	145 (3.6)	-	0.79 (0.29-2.17); 0.644
**Deep venous thrombosis**	86 (2.1)	-	1.08 (0.54-2.14); 0.833
**Arterial thrombosis**	233 (5.8)	0.96 (0.53-1.75); 0.899	-
**Acute myocardial infarction**	68 (1.7)	0.26 (0.04-1.89); 0.184	-
**Acute cerebrovascular insult**	111 (2.8)	0.83 (0.34-2.07); 0.694	-
**Bleeding**	322 (8)	1.34 (0.85-2.11); 0.213	1.84 (1.24-2.74); 0.003
**Gastrointestinal bleeding**	133 (3.3)	1.46 (0.76-2.83); 0.256	1.8 (1.0-3.25); 0.049
**Corticosteroid therapy**	2792 (69.6)	1.25 (0.91-1.71); 0.163	0.73 (0.56-0.97); 0.028
**Intensified corticosteroid therapy**	1157 (28.8)	2.14 (1.62-2.83); <0.001	0.91 (0.68-1.23); 0.535

*Abbreviations: OR – odds ratio; CI – confidence interval; ECOG – Eastern Cooperative Oncology Group; MEWS – modified early warning score.

†n (%) or median (interquartile range).

ROC curve analysis showed the cut-off level for D-dimer on admission with the best discriminatory properties for venous thromboses prediction to be >2.19. The sensitivity was 67.9% and the specificity was 66.3% (AUC 0.716, *P* < 0.001). Twelve percent of patients above and 3.2% of patients below this level experienced venous thrombosis. Considering the level of care required, patients treated in the intensive care unit had significantly higher frequencies of both DVT (3% vs 1.9%) and PE (5.8% vs 3%) (Figure 1B). When considering VTE on admission and subsequent events, there was a similar proportion of intensive care unit use (42.6% and 31.2%, respectively). The minority of events were detected after intensive care unit admission (6.9% of pulmonary embolisms and 14% of DVT). VTE were surprisingly associated with longer hospital survival (VTE 6% vs 4.1% for surviving vs dying; mortality 27.6% vs 36% in patients with and without VTE). However, when considering only patients with defined VTE on admission, no significant relationship with in-hospital survival was present (*P* = 0.961), which suggests that subsequent events were evaluated dominantly in survivors. Furthermore, VTE were more common in patients receiving pharmacologic thromboprophylaxis (5.8% vs 2.5%), which suggests that events were probably less likely to be evaluated early during the pandemic when the LMWH use was not established as a standard of care.

In the multivariate logistic regression analysis performed by backward approach and including univariately significant associations, parameters that remained mutually independently associated with venous thromboses were metastatic malignancy (OR 2.58; *P* = 0.004), known thrombophilia (OR 10.13; *P* = 0.021), lower MCHC (OR 0.98; *P* = 0.036), D-dimer >2.19 (OR 3.49; *P* < 0.001); lower LDH (OR 0.99; *P* = 0.034), longer duration of disease on admission (OR 1.05; *P* < 0.001), bilateral pneumonia (OR 2; *P* = 0.024), longer duration of hospitalization (OR 1.03; *P* < 0.001), and immobilization for at least one day (OR 4.34; *P* = 0.001).

### Factors associated with arterial thromboses

Considering general characteristics, comorbidities, and drugs in chronic therapy, arterial thromboses were significantly associated with older age (median 77 vs 73 years); higher Charlson Comorbidity Index (median 6 vs 4); active smoking (thrombosis 9.9% vs 5.5%), higher number of drugs in chronic therapy (median 7 vs 5), arterial hypertension (thrombosis 6.7% vs 3.8%), diabetes mellitus (thrombosis 7.5% vs 5.1%), hyperlipoproteinemia (thrombosis 7.8% vs 5.2%), congestive heart failure (thrombosis 9.4% vs 5.1%), atrial fibrillation (thrombosis 9% vs 5.1%), coronary artery disease (thrombosis 12.6% vs 4.6%), peripheral artery disease (thrombosis 19.2% vs 4.8%), history of myocardial infarction (thrombosis 12% vs 5.2%), history of cerebrovascular insult (thrombosis 15.4% vs 4.5%), inflammatory bowel disease (thrombosis 13% vs 5.7%), use of aspirin (thrombosis 11.2% vs 4.5%) and statin (thrombosis 8.7% vs 4.9%) in chronic therapy, and absence of active malignant disease (thrombosis 3.5% vs 6.1%) or metastatic malignant disease (thrombosis 2.5% vs 6.1%).

Considering laboratory parameters on admission, arterial thromboses were significantly associated with lower C reactive protein (CRP; median 63.8 vs 90.6 mg/L) and higher D-dimer (median 1.73 vs 1.39 mg/L FEU). Considering COVID-19 severity and hospitalization-related parameters, arterial thromboses were significantly more frequent in patients with less severe COVID-19 on admission (thrombosis 5.4% vs 7.8% among severe or critical vs mild or moderate COVID-19), shorter disease duration on admission (median 2 vs 5 days), worse ECOG status on admission (median 3 vs 3 points), other infection on admission (thrombosis 8.3% vs 5.4%), mechanically ventilated patients (thrombosis 8.1% vs 5.3%), patients immobilized for at least one day (thrombosis 7.8% vs 0.9%), patients immobilized for ≥7 days (thrombosis 7.5% vs 4.5%), and patients with lower limb paralysis (thrombosis 10.6% vs 3.7%). Arterial thrombotic events were significantly associated with bleeding (thrombosis 13.3% vs 7.7%), with a similar proportion of thrombotic events occurring before and after bleeding (6.4% vs 6.9% of all arterial thrombotic events). It was not significantly associated with LMWH thromboprophylaxis.

Myocardial infarction and peripheral thromboses were significantly associated with intensive care unit use, whereas patients with CVI and mesenterial thromboses had similar frequency of intensive care unit use as non-arterial thrombosis patients. A minority of events of each subtype occurred after intensive care unit admission (2.9% of all myocardial infarctions, 20.5% of all cerebrovascular insults, 9% of all peripheral embolisms, 20% of mesenteric thromboses). The presence of arterial thrombosis was significantly associated with inferior survival (thrombosis 4.2% vs 8.7% for surviving vs dying; mortality 53.2% vs 34.5% in patients with and without arterial thrombosis), with patients with later occurring events having significantly higher mortality than patients presenting with arterial thrombosis on admission (80.5% vs 38.4%).

In the multivariate logistic regression analysis performed by backward approach and including univariately significant associations, parameters that remained mutually independently associated with arterial thromboses were mild or moderate COVID-19 on presentation (OR 0.35; *P* < 0.001), higher Charlson Comorbidity Index (OR 1.12; *P* = 0.012), coronary artery disease (OR 1.84; *P* = 0.011), peripheral artery disease (OR 2.98; *P* < 0.001), history of cerebrovascular insult (OR 2.18; *P* = 0.001), aspirin use (OR 1.71; *P* = 0.011), lower CRP (OR 0.99; *P* = 0.024), better ECOG functional status on admission (OR 0.83; *P* = 0.034), intensive care unit use (OR 2.25; *P* < 0.001), immobilization for at least one day (OR 7.37; *P* < 0.001), absence of hyperlipoproteinemia (OR 0.44; *P* < 0.001), and absence of metastatic malignancy (OR 0.33; *P* = 0.033).

## Discussion

Our data confirm a substantial thrombotic burden associated with COVID-19, with 5.3% patients experiencing a venous and 5.8% patients experiencing an arterial thrombotic event. Although both types of events were associated with the need for intensive care, they differed in the timing of presentation, association with COVID-19 severity, and other clinical features. Furthermore, our data suggest that VTE events might be underrecognized in our cohort.

COVID-19-associated prothrombotic state, often termed “thromboinflammation,” (13) does not always manifest as or is not recognized as a full developed thrombotic event. Patients with severe COVID-19 develop excessive inflammation and a spectrum of coagulation, platelet, and endothelial abnormalities that contribute to a prothrombotic state. These abnormalities include elevated D-dimer, increased fibrinogen and factor VIII activity, platelet activation, endothelial cell invasion and endothelial dysfunction, blood stasis, and other (17-19). LMWH administration in prophylactic doses may prevent thrombosis, reduce systemic and pulmonary inflammation, and limit viral invasion (20-22). Since LMWH thromboprophylaxis improves the survival of hospitalized COVID-19 patients, it has become a standard of care. There is insufficient evidence to recommend the use of higher than the prophylactic doses of anticoagulation for routine VTE prophylaxis in these patients. Nevertheless, COVID-19 patients who are suspected of having a venous thromboembolic event or rapid respiratory deterioration are often treated with full therapeutic doses of anticoagulation. Thromboembolic complications can be already present at the initial hospital admission as many ultimately hospitalized patients are initially being managed on an ambulatory basis in early stages of disease when no routine thromboprophylaxis is recommended. Upon hospital admission, decision whether to fully anticoagulate a deteriorating patient without proven thromboembolism or withdraw the pharmacologic thromboprophylaxis in a patient experiencing bleeding complication is an everyday dilemma of physicians treating COVID-19 patients, left to individual clinical judgment since life threatening complications may occur in both cases. A large majority of our patients received pharmacologic thromboprophylaxis (86%). We were unable to fully identify the proportion of patients who received intermediate or full therapeutic LMWH doses in the current study and put LMWH treatment intensity in the context of the current paper. The reason for this was that thromboprophylaxis was provided according to the treating physician’s discretion and usually varied in intensity in the same patients during hospital stay. No routine arterial thrombosis prophylaxis is currently recommended, but antiplatelet therapies were usually continued if secondary prevention was being instituted before COVID infection for other indications.

The incidences of VTE, PE, and DVT in our patients were 5.3%, 3.6%, and 2.1%, respectively. Similar incidences were reported in other retrospective cohorts (23). There is considerable variation in the frequency of VTE in the literature, and VTE events develop despite the use of pharmacologic thromboprophylaxis. Moreover, thromboprophylaxis might ameliorate and mask the clinical presentation during duration of therapy (24). The VTE incidence/detection rate seems to depend on COVID-19 severity in investigated cohorts and whether active screening for VTE was performed. A large retrospective analysis of over 3000 hospitalized patients reported PE incidence of 3.2% and DVT incidence of 3.9% in the overall cohort, and 6.2% and 9.4%, respectively, in ICU treated patients (23), which is comparable to our results. However, studies that systematically assessed DVT by bilateral leg ultrasound reported a very high frequency of mostly asymptomatic DVT, ranging from 11.9% to 22.5% among hospitalized ward patients (25-27) and from 65% to 69% among ICU patients on admission (28,29). We performed a similar study in our patients who were hospitalized for a long time, before hospital discharge. Among 102 patients without clinically overt DVT, asymptomatic DVT was detected in 25.5% patients despite all patients receiving thromboprophylaxis. The frequency of DVT reached 60% in mechanical ventilation survivors (24), which confirmed previously published reports. Thus, the more thromboses are searched for, the more they are discovered.

Known thrombophilia is a reported predictive factor for both asymptomatic thromboses (24) and VTE in general, as shown in the current study. However, the definition of thrombophilia status is vague and hard to quickly obtain, even if patients were previously tested or are currently suspected. In the current study, known thrombophilia was very low in prevalence and was considered present if the patient had positive both family and personal history of thrombosis, had pre-recorded known hereditary thrombophilia (patients in our study had either factor V Leiden, factor II G20210A mutations, and one patient had known factor XII deficiency), or had a highly thrombogenic acquired condition (heparin-induced thrombocytopenia).

Higher COVID-19 severity and features of stronger inflammation have been recognized as predictors of VTE in several cohorts (30-32) and in the current study, highlighting the interconnected pathways of hyperinflammation and thrombosis. Lower MCHC is a red blood cell index reflecting hemoglobin content and the quality of erythropoiesis. It is an independent predictor of VTE not overlapping in predictive properties with other investigated parameters. Lower MCHC is usually considered a marker of higher severity of inflammatory condition or of an underlying iron deficit/restriction in the context of anemia of chronic disease. MCHC was reported to decrease during COVID-19 course and with more severe disease, despite still remaining in the reference range in most patients (33). Among recovered COVID-19 patients, the nadir MCHC levels, together with mean corpuscular volume (MCV), independently predicted disease progression (33).

The current study observed an association of VTE with improved survival. This finding could be explained by the fact that imaging studies were performed dominantly in survivors and patients sufficiently stable to undergo radiologic procedures, which introduced a selection bias. COVID-19 patients are dependent on the oxygen source and might suffer from multiorgan failure due to acute inflammatory state associated with acute SARS-CoV-2 infection. LMWH treatment in prophylactic, but often intermediate or full therapeutic doses, masks the overt clinical presentation of VTE. VTE then might exert its full clinical potential after hospital discharge, when patients receive no or limited dose and duration of anticoagulant therapy. Considering these observations, VTE events are underdiagnosed in retrospective studies of COVID-19 patients, such as this one, where only clinically evident events were documented. Current guidelines do not argue either for or against routine DVT ultrasound screening in COVID-19 patients. In our opinion, an accumulating pool of evidence calls for the change of this recommendation, as a more intensive diagnostic approach is probably needed to tailor optimal treatment for COVID-19 patients.

High rates of arterial thrombotic events associated with COVID-19 have been well-recognized (6,34,35). Arterial thromboses often require urgent treatment and, due to dramatic clinical presentation, are more readily recognized. In contrast to PE, whose signs and symptoms overlap with those of severe COVID-19 and might be attenuated with thromboprophylaxis, arterial thromboses result in acute ischemia of the affected vascular beds and are less likely to be misinterpreted in the context of COVID-19. Arterial thromboembolism might also be the first manifestation of COVID-19 (36,37). These patients presented earlier than other patients in our study. A subset of these patients developed respiratory symptoms later during the disease course. In these patients, direct endothelial or vascular injury might be a major factor leading to occlusive atherothrombosis. Since our center is a tertiary-level COVID-19 institution, these patients may also be over-represented in the current study due to referral. This is a probable reason why the frequency of arterial thromboses was higher than in previous reports (38,39).

Clinical parameters associated with venous and arterial thrombotic events differed substantially. VTE in our data set were independently predicted by more severe COVID-19 on admission, longer disease duration, greater extent of lung involvement, and factors associated with VTE (metastatic malignancy, known thrombophilia, immobilization, higher D-dimer on admission). On the contrary, arterial thrombotic events were independently associated with less severe COVID-19 on admission, shorter duration of symptoms, lower CRP, better functional status on admission, higher comorbidity burden, established atherosclerotic disease, aspirin use, and other. These findings are in line with the majority of arterial events occurring at admission, earlier during the disease course when full clinical presentation of COVID-19 did not have time to develop. Furthermore, patients developing arterial thrombotic events seem to be predisposed to these events due to cardiovascular disease burden. High mortality associated with arterial thrombotic events in the context of COVID-19, especially if events occurred later during the hospitalization, highlights the heavy negative prognostic impact of COVID-19 pneumonia on the survival of patients with acute arterial thrombotic events, which seems to be worse than in non-COVID-19 patients (12,40).

Finally, infection-associated vascular injury has long been described in many types of infections, especially after sepsis and septic shock (14,41). Non-COVID-19 viral respiratory infections, including viral influenza, confer excess thrombotic risk after hospital discharge. Nevertheless, the risk in COVID-19 seems to be 3-fold higher than in non-COVID-19 respiratory illness (42). Since we do not have published national or regional data about thrombosis burden in COVID-19 and non-COVID-19 respiratory illness, future studies should elucidate whether and to what extent thromboembolic complications differ between these diseases. Thus future, preferably prospective studies, with active screening for events, are needed.

Data reported in our article represent a tertiary-level center experience and contemporary thromboprophylaxis and diagnostic standards. The main limitations of our work are the single-center experience and retrospective study design. We could not evaluate the contribution of LMWH thromboprophylaxis intensity due to inter- and intra-individual heterogeneity in different patients. An additional factor was that more intensive thromboprophylaxis was given to deteriorating patients who were more intensively treated and were more likely to experience a thrombotic event. Selection bias associated with the evaluation of VTE in sufficiently clinically stable, surviving patients limits the assessment of the recorded event rates and associated risk factors. No causal relationship can be inferred from the presented data due to the retrospective study design. Nevertheless, large sample size and real-life patient experience representative of contemporary treatment practices enable us to draw clinical lessons from our data.

In conclusion, among hospitalized COVID-19 patients, venous and arterial thromboses seem to be highly prevalent, especially among ICU treated patients, and may differ in the timing of presentation, association with COVID-19 severity, and other clinical characteristics.
